# New Insights about Pulmonary Epithelioid Hemangioendothelioma: Review of the Literature and Two Case Reports

**DOI:** 10.1155/2017/5972940

**Published:** 2017-08-14

**Authors:** Romeu Duarte Mesquita, Marta Sousa, Carmen Trinidad, Eugénia Pinto, Iosu Antón Badiola

**Affiliations:** ^1^Department of Radiology, Centro Hospitalar de Entre o Douro e Vouga, Santa Maria da Feira, Portugal; ^2^Department of Radiology, Hospital Povisa, Vigo, Spain; ^3^Department of Pathology, Centro Hospitalar de Lisboa Central, Lisboa, Portugal; ^4^Department of Pathology, Hospital Povisa, Vigo, Spain

## Abstract

Pulmonary epithelioid hemangioendothelioma (PEH) is a rare neoplasm of vascular origin. There are three different major imaging patterns identified in thoracic manifestation of epithelioid hemangioendothelioma: (1) multiple pulmonary nodules; (2) multiple pulmonary reticulonodular opacities; and (3) diffuse infiltrative pleural thickening. Radiographically, presence of bilateral multiple nodules is the most common pattern of presentation. The diagnosis is made on the basis of histopathological findings and confirmed by positive immunohistochemistry staining. Although the prognostic factors for PEH have not yet been well established, a better prognosis is usually associated with the multinodular pattern. We report two different imagological presentations of this rare disease, based on two institutional experiences, along with a review of the relevant literature.

## 1. Introduction

Pulmonary epithelioid hemangioendothelioma (PEH) is a rare neoplasm of vascular origin. It is a low-to-intermediate grade malignant tumor, of borderline malignancy and a clinical course usually between hemangioma and angiosarcoma. Unfortunately, there is no clear definition of malignant PEH. The term “epithelioid hemangioendothelioma” was first introduced by Weiss et al. as a soft tissue vascular tumor of borderline malignancy [1].

However, the lesion was originally described in 1975 by Dail et al. as an “intravascular bronchoalveolar tumor,” initially believed to be an aggressive form of the bronchoalveolar cell carcinoma [2].

Epithelioid hemangioendothelioma (EH) is classified as a vascular tumor because the neoplastic cells have characteristics in common with normal, nonneoplastic endothelial cells [3]. EH can behave in a locally aggressive fashion, with metastases being rare [4].

Corrin et al., using immunohistochemical techniques, demonstrated the presence of malignant cells deriving from a lineage capable of differentiation along endothelial lines [5]. Subsequently, Weldon-Linne et al., using electron microscopy, confirmed these results and revealed diffuse cytoplasmic staining of the tumor cells with a factor VIII-related antigen [6].

Histologic characteristics of EH include epithelioid cells with abundant eosinophilic cytoplasm in a fibromyxoid stroma, some of them with intracytoplasmic vacuoles, having a signet ring-like appearance. These features can, however, be also found in primary adenocarcinoma, mesothelioma, and large-cell lymphoma, so immunohistochemical analyses, especially those for the endothelial markers, are a mainstay [7–10].

The 2015 WHO Classification of Tumors of the Lung, Pleura, Thymus and Heart [11] recently published, with numerous important changes from the 2004 WHO classification, has a significant change involving recognition of usefulness of* WWTR1-CAMTA1 *fusions in diagnosis of pulmonary EH. The new information regarding this tumor is recognition of a translocation involving the* WWTR1 *and* CAMTA1 *genes and prognostic factors [12].

This update was based on the recent recognition that a recurrent t(1;3)(p36.3;q25) chromosomal translocation is characteristic of EH. Additionally, the genes involved in the EH translocation were identified, namely, WWTR1 (or TAZ) and CAMTA1, resulting in a transcription factor, WWTR1-CAMTA1 fusion protein. Also significant is the lack of CAMTA1 and WWTR1 rearrangements in* epithelioid hemangioma*, a benign vascular tumor that can be misdiagnosed as EH [13–17].

Tanas et al. [18] suggest that the* WWTR1*/*CAMTA1 *gene fusion plays a pivot role in the tumor biology and can act as a transcription factor that ultimately may represent a therapeutic target for EH-specific drugs. Recently, Antonescu et al. [19, 20] report a novel subset of EH occurring in a specific group of young adults, where they found a distinct phenotype with YAP1-TFE3 fusions but similar morphologic features. Although further studies [21] confirm these results others question the utility of immunohistochemical YAP1-TFE3 fusion detection [7, 14].

Another hypothesis for the pathogenesis of this neoplasm, reported in the literature, mentions a possible causal relationship between chronic* Bartonella *infection and the development of this rare vascular tumor in immunocompetent patients [22].

Some authors also believe in a hormonal potential significance in this disease because the greater incidence is observed in women; also, hormone receptor expression has been reported in the literature [23, 24] and a recent case report describes recurrence of EH during pregnancy [25].

All these data highlight the current emerging development about the knowledge concerning biochemical and clinical behaviour of EH in general and PEH in particular and can lead to greater understanding of the molecular pathways in the tumor and great advances in potential therapies.

EH remains, however, a little-understood disease and one of the main reasons for that is the fact that the prevalence of EH is reported to be less than one in 1 million [26].

## 2. Case 1

**Figure 1:**
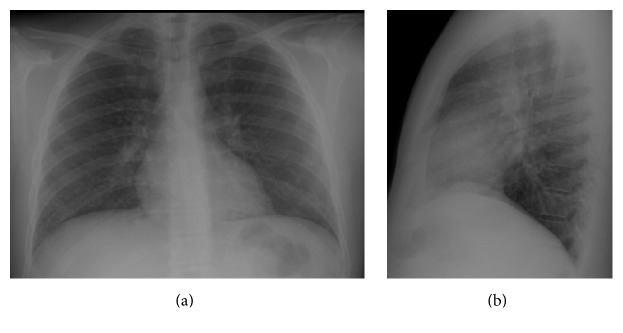
Posteroanterior (a) and lateral (b) chest X-rays. The chest radiography showed a diffuse nodular pulmonary pattern in both lungs.

A 35-year-old male, nonsmoker, without symptoms and with no prior history of lung diseases had a routine chest radiograph that showed alterations. His past medical history was not significant, without occupational exposures or family history of clinical relevance. The physical examination was unremarkable. Chest X-ray demonstrated the presence of a diffuse and bilateral micronodular infiltrate (Figure 1).

**Figure 2:**
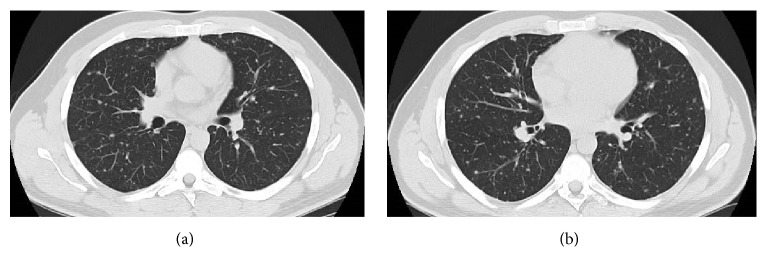
Chest CT (a and b). Multislice chest CT-images on lung window showed innumerable, well-defined, small, round, and noncalcified pulmonary nodular opacities, scattered in both lungs. These nodules were present in all pulmonary lobes and adjacent to bronchioles and medium/small vessels.

Serum levels of neoplastic markers and angiotensin converting enzyme were within normal values. The pulmonary function tests were also in the normal range. The bronchoscopy performed with bronchoalveolar lavage, brushing, and transbronchial biopsies were nondiagnostic. Chest high resolution (HR) computed tomography (CT) scan showed multiple, bilateral small pulmonary nodules (with diameters inferior to 10 mm) (Figure 2).

**Figure 3:**
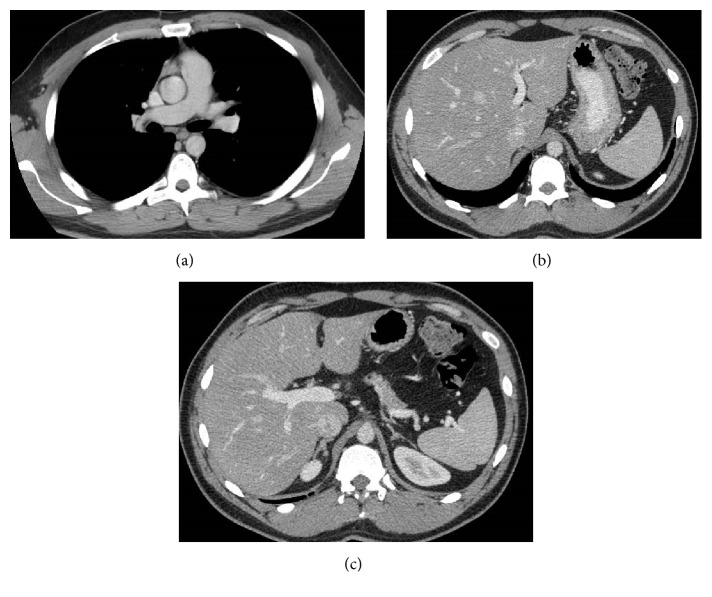
Contrast-enhanced multislice CT of the chest (a) on mediastinal window showed no thoracic lymphadenopathy. Multislice abdominopelvic CT scans (b and c) with intravenous contrast administration showed no significant findings, excluding focal liver or splenic lesions.

These nodules were present in all pulmonary lobes and adjacent to bronchioles and medium/small vessels. There were no enlarged lymph nodes, pleural effusion, or other relevant pulmonary changes. Abdominal and pelvic CT revealed no malignant lesions (Figure 3).

**Figure 4:**
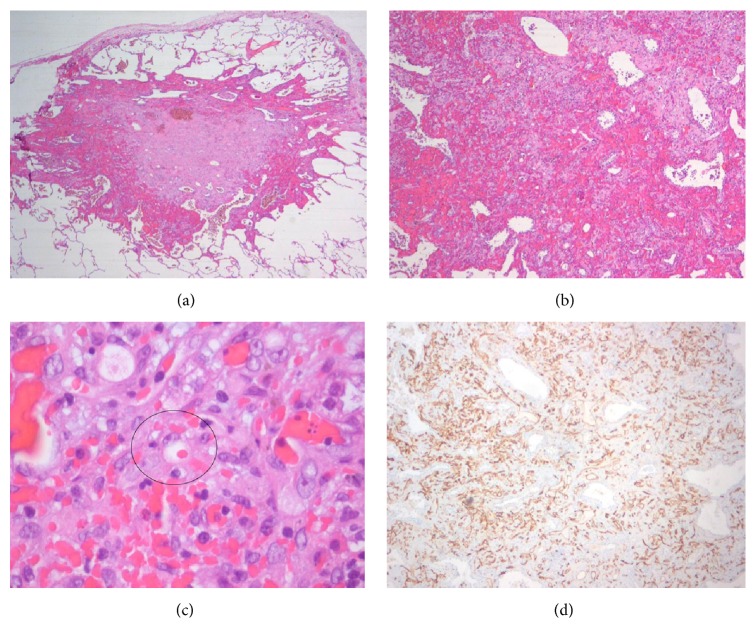
Histopathologic findings of hemangioendothelioma. (a) Low magnification of the specimen obtained from pulmonary wedge resection reveals a subpleural nodule that extends to adjacent alveoli, with vascular proliferation in the peripheral zone and areas of recent and old hemorrhage. (b) Neoplastic nodule showing tumor cells with an eosinophilic stroma at the periphery. The lesion contains blood filled spaces and respiratory epithelium “trapped” within the lesion is also shown. (c) Higher magnification of the tumor reveals vacuolation of some of the tumor cells, representing primitive angiogenesis. One of these epithelioid cells can be observed with intracytoplasmic lumen and an erythrocyte inside, indicating their vascular nature. (d) Immunostaining for CD34 revealed positivity of the neoplastic cells (brown colour), confirming the endothelial lineage of the tumor.

Radiological skeletal study also showed no bone involvement. Surgical lung biopsy was performed and histological examination of the nodules revealed epithelioid cells with eosinophilic cytoplasm and intracytoplasmic vacuolization. On immunohistochemical studies, the tumor cells were immunoreactive to CD34 and negative for cytokeratin (Figure 4).

Because the progression was slow, follow-up was suggested. In agreement with his decision, close follow-up with chest CT was planned.

He still has no symptoms and has survived for 48 months following the initial diagnosis.

## 3. Case 2

**Figure 5:**
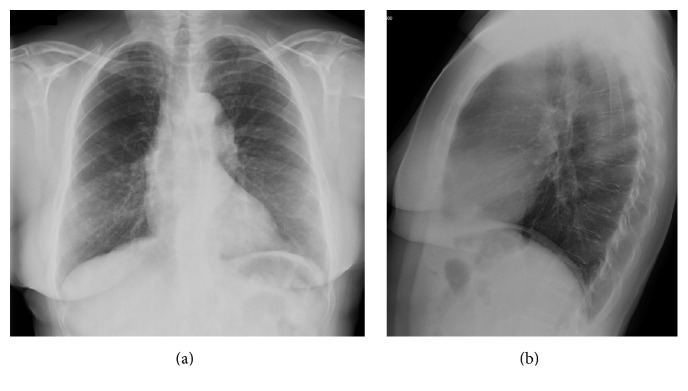
Posteroanterior (a) and lateral (b) chest X-rays. In a routine chest radiography 2 years before presentation in the ER it was difficult to detect the lesion in the left lower lung.

A 67-year-old woman, nonsmoker, presented in the emergency department with a complaint of mild productive cough for 2 months with hematic sputum in the last 24 hours. She also reported progressive left-sided dorsal pain over 1-month duration. Her past medical history revealed a previous diagnosis of deep vein thrombosis in 2012. In a routine chest radiograph made 2 years previously, it was hard to depict any pulmonary pathology (Figure 5).

**Figure 6:**
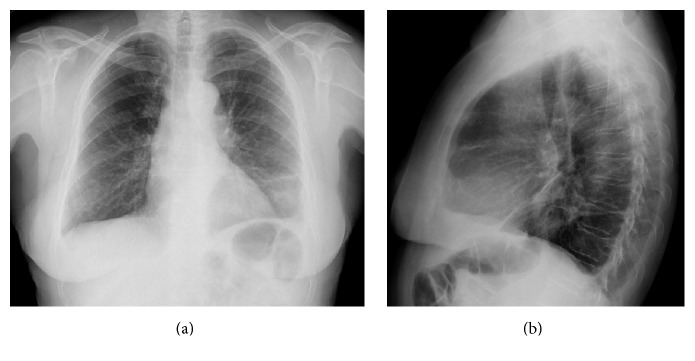
Posteroanterior (a) and lateral (b) chest X-rays. The chest radiography showed a peripheral lesion in the left lower lung and a small volume of pleural effusion on the same side.

Her family history was unremarkable. There were no other specific abnormalities on physical examination. The laboratory findings were within normal range. Chest X-ray demonstrated the presence of a poorly defined opacity in the left lower lung and a blunt costophrenic angle in the left side (Figure 6).

**Figure 7:**
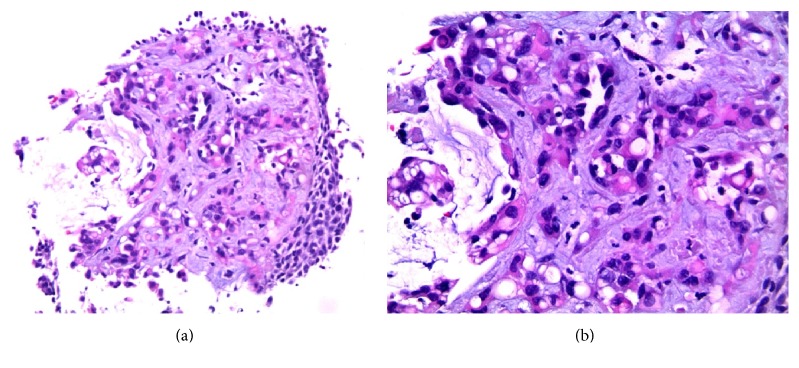
Transbronchial biopsy of a pulmonary mass (H-E). (a) 10x. (b) 20x. Vascular proliferation and endothelial cells, frequently with citoplasmatic vacuolization, in a fibromyxoid stroma, with bronchial surface epithelial lining.

Spirometry showed forced vital capacity (FVC) of 1.59 (69.1%), forced expiratory volume in 1 second (FEV1) of 1.16 (60.7%), FEV1/FVC of 76%, and diffusing capacity divided by the alveolar volume (DLCO/VA) of 103.3%. Extensive pulmonary workup for pulmonary nodules including bronchoscopy, bronchoalveolar lavage (BAL), and transbronchial lung biopsy was completed. Results were negative for infectious or rheumatologic processes. The transbronchial biopsy showed atypical cellular infiltrate with immunohistochemical expression very suggestive of proliferative lesion of vascular origin (Figure 7).

**Figure 8:**
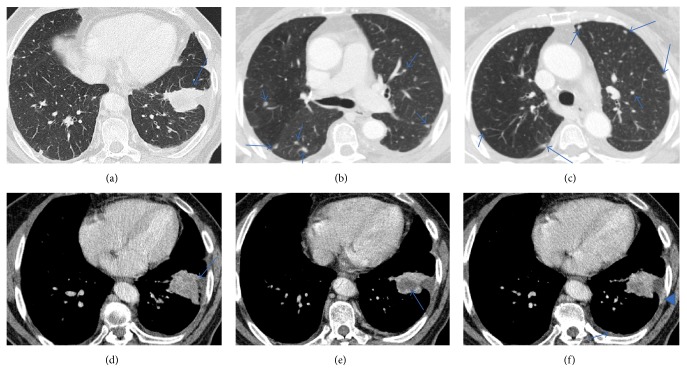
Chest CT. Multislice chest CT-images on lung window (a, b, and c) showed a lung mass with irregular borders (a); they also showed multiple small, round, and noncalcified pulmonary nodular opacities, scattered in both lungs (b and c). On mediastinal window (d, e, and f) it is possible to better identify the pleural invasion (d) and the necrotic areas in lesion (e), retrospectively, and valorization is hard when the exam was performed, and we can recognize areas of enhancement in the left pleura, more nodular (arrow) or linear (arrowhead) (f).

A chest CT scan revealed the presence of a lung mass in the parenchyma of the left inferior lobe (LIL), with necrotic component and in contact with the parietal pleura and diaphragmatic pleura, with associated partial atelectasis of the same lobe and coexisting multiple nodules scattered in both lungs (Figure 8); a single hilar ipsilateral lymphadenopathy was identified and no mediastinal lymphadenopathy or significant pleural effusion were present at presentation.

**Figure 9:**
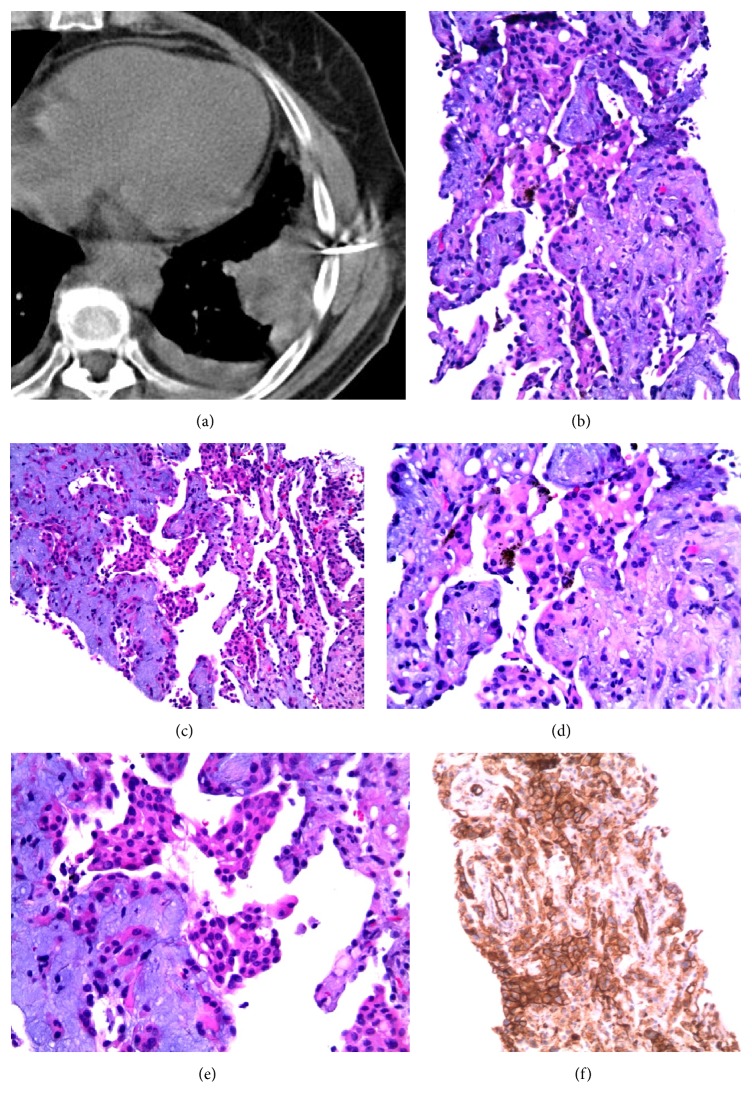
20 G Tru-Cut biopsy of the lung mass (a). H-E (b–e) images show vascular proliferation with epithelioid cells in a fibromyxoid stroma and presence of haemosiderophages ((b, c) 10x; (d) 20x). Some of the epithelioid cells show vacuolation (e). Immunostaining for CD31 revealed positivity of the neoplastic cells (brown colour), confirming the endothelial lineage of the tumor.

Patient underwent a first chest CT-guided lung biopsy that revealed intense fibrosis and focal changes suggestive of proliferative vascular lesion. She repeated the lung biopsy that showed the presence of atypical epithelioid-like tumor cells (Figure 9). Immunohistochemical studies were positive for CD31, CD34, and factor VIII. These results were consistent with the diagnosis of PEH.

She underwent a FDG PET/CT (not shown) that showed areas of increased uptake of FDG, including the pulmonary mass in the LIL as a hypermetabolic lesion and the left hilar lymphadenopathy. There was no abnormal FDG uptake in the small pulmonary nodules

**Figure 10:**
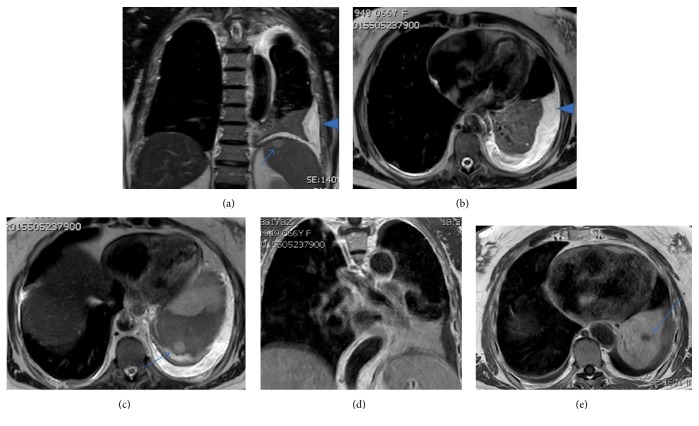
Thoracic MR images. Fluid sensitive sequences (a–c) show the presence of left pleural effusion (arrowhead) and also the nodular lesions in the spleen, which do not correspond to cysts (arrows). T1 weighted images with contrast (d and e) show the pulmonary mass and hypodense zones representing necrotic areas (arrow).

One thoracic magnetic resonance (MR) with paramagnetic contrast, done two months later, demonstrated a left pleural effusion but did not show unequivocal abnormal pleural enhancement compatible with pleural metastases (Figure 10).

Three months later, under general anaesthesia, the surgeons performed a wedge resection by thoracotomy that also revealed multiple small pleural implants, both in visceral and in parietal pleural lesions, which were biopsied. Tumor specimens acquired showed pathological features of EH.

After a scheduled oncology visit the patient began treatment with six cycles of chemotherapy with carboplatin/paclitaxel, which changed to doxorubicin/cyclophosphamide.

**Figure 11:**
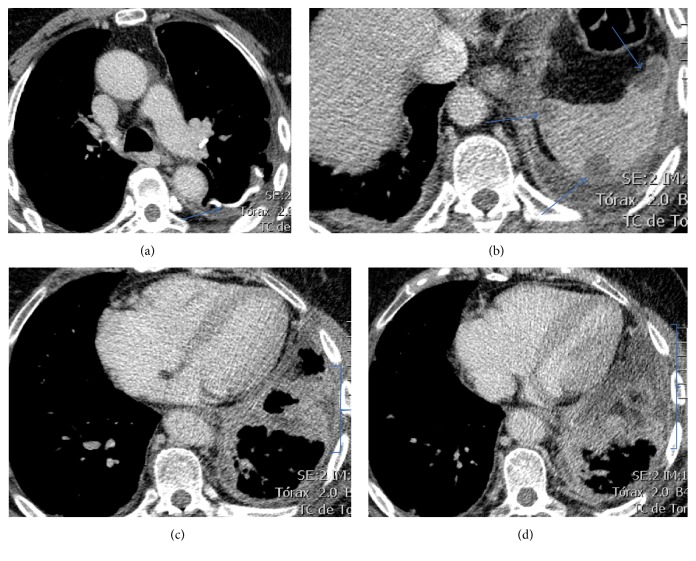
Chest CT. Multislice chest axial CT-images on mediastinal window show (a) signs of previous atypical wedge resection in the left lower lobe (arrow); (b) more nodular lesions in the spleen (arrows); (c and d) more pronounced and diffuse pleural invasion (brackets).

Another chest CT, three months after the initial CT, showed rapid progression of the disease depicted by increase in number of the pulmonary nodules and the pleural lesions. Also, showing increment in diameter, the splenic nodular lesions were compatible with metastases (Figure 11).

The patient returned to emergency department, seven months after the first visit, with progressive dyspnea.

**Figure 12:**
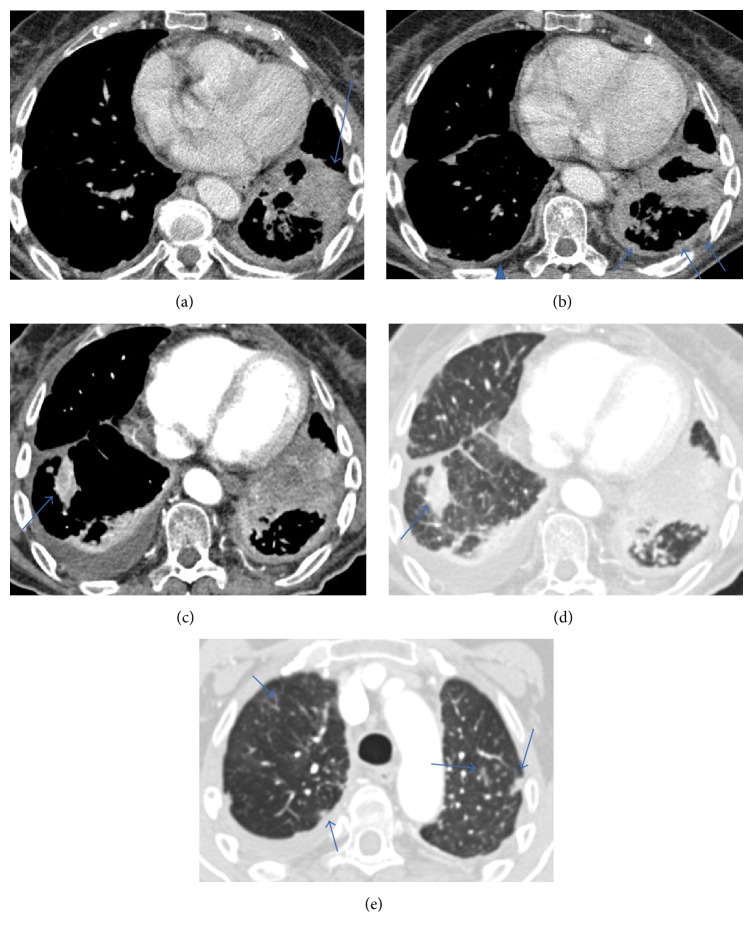
Chest CT. Multislice chest axial CT-images on mediastinal window (a, b, and c) show increase in size of the main lesion (a) and pleural metastases in the left side (arrows) and in contralateral side (arrowhead); a new lesion in the right lung is observed (arrow in (c)). Multislice chest axial CT-images on lung window (d and e) show the new contralateral lesion (arrow in (d)) and the parenchymal pulmonary nodules did not vary significantly in size from the previous studies (arrows in (e)).

A chest CT made at this time showed advance disease with a new lesion appearing in the contralateral pulmonary parenchymal and growth of the primary lesion in the left lung, illustrating the aggressive behaviour of the disease. The pulmonary nodules, however, did not show significant increment in size despite the completely different evolution seen in the lesion of the lower left lung (Figure 12).

**Figure 13:**
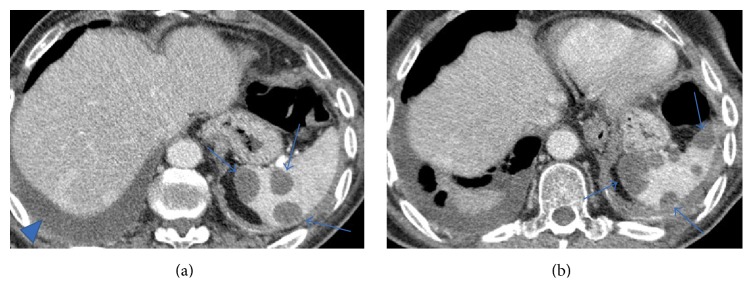
Multislice chest CT. Axial images on mediastinal window at the pulmonary bases (a and b) show increase in size and number of the nodular spleen lesions (arrows). A pleural effusion in the right side is also apparent (arrowhead).

Unlike this, the spleen showed an increase in number and size of the nodular lesions in his parenchyma. Although we did not have pathologic confirmation, because the patient did not have any other primary neoplastic lesion, these features reinforce the probable diagnosis of metastases from the PEH (Figure 13).

Upon her readmission, 2 weeks later, the patient succumbed to her condition due to respiratory failure.

## 4. Discussion

Using the International Hemangioendothioma, Epithelioid Hemangioendothelioma, and Related Vascular Disorders (HEARD) Registry, an Internet database with data of 206 patients, Lau et al. [15] found that the most commonly affected organs were liver and lung, and single organ involvement occurred in more than 60% of patients. The most common presentations were liver alone (21%), liver plus lung (18%), lung alone (12%), and bone alone (14%).

There is still no characteristic clinical or biological marker for PEH. PEH typically occurs among young patients and is more common in women than in men and many patients are asymptomatic at presentation, so it is often an incidental finding on imaging studies. Other possible symptoms can be minor or nonspecific pulmonary symptoms, such as chest pain, effort dyspnea, cough, and sputum [8, 14, 26–32]. There are sporadic reported cases of atypical symptoms related to unusual clinical course and/or extraordinary aggressiveness [33].

The prognosis is very variable, with survival ranging from less than one year up to 30 years [34]. Slow progression or growth for long periods and spontaneous regression may occur, especially in asymptomatic patients [27]. According to Lau et al. analysis, the 5-year overall survival is 73% [15]. The mortality for lung disease, with a minimum of follow-up of 4 years, was reported to be 65% [1].

The prognostic factors for PEH have not yet been well established. Pulmonary symptoms and, specially, pleural hemorrhagic effusions and hemoptysis are generally considered the worst prognostic factors [28]. Symptomatic patients and presence of pleural effusion were found to be independent predictors of survival. Other significant risk factors for PEH are male gender, presence of cough, chest pain, multiple unilateral nodules, metastases to more than one site, and lymph node metastases [26].

Another study using cases from International HEARD Support Group found that although concurrent multiorgan involvement is common at the time of diagnosis, no specific organ or combination of organ involvement differentially affected survival, and there were no differences in patient's survival with single versus multiple organ involvement in their cohort [15]. They also confirmed that hemoptysis and pleural effusion or other signs of uncontained tumor growth have prognostic value and involve worse survival. These authors also categorized the patients into two different patterns of disease at presentation (A and B) based on presence or absence of lesions with discrete/defined borders, with pattern B in the chest including pulmonary infiltrates, pleural effusion, extrapulmonary thoracic disease, and the symptom of hemoptysis and found a strong predictive prognostic value.

In terms of clinical features, it seems that the prognosis of PHE reflects primarily the vascular aggressiveness markers of hemorrhagic symptoms.

Although there are controversies regarding the multicentric or metastatic nature of EH, it can affect many organs simultaneously or sequentially, and lungs and liver are the two organs most frequently involved [15, 30, 35]. Although PEH is capable of producing regional and distant metastases, the metastases occur less frequently than in conventional angiosarcoma [26].

Dissemination of PEH can occur through blood vessels and lymphatics and within the pleural cavity [36]. Although rare, when the primary location is the lungs, distant metastases to the liver, skin, kidney, spleen, and retroperitoneum can occur and have been reported in the literature [2, 27, 28, 37–41]. Weiss et al. [1] reported that metastatic disease occurs in approximately 15% of patients with lesions affecting the lung.

Although it is unclear if the expression of CD44 has a major role in PEH invasiveness and metastases (because the number of patients tested is limited) Yi et al. [42] suggested the potential of intravascular invasion in a patient presenting with pulmonary thromboembolic disease and pulmonary hypertension.

Nevertheless, in patients with also hepatic involvement, knowledge of the CT manifestations in the liver may be helpful to narrow the differential diagnoses.

EH in the thorax can involve not just the lungs but also the pleurae and the mediastinum [14, 31, 43–45]. There are three major different patterns of CT findings identified in thoracic manifestation of EH [46–48]: (1) multiple pulmonary nodules; (2) multiple pulmonary reticulonodular opacities; and (3) diffuse infiltrative pleural thickening.

Radiographically, bilateral multiple nodular nodules are the most common presentation [27, 49]. The presence of multiple discrete pulmonary perivascular nodules with well- or ill-defined margins in both lungs on chest radiographs or CT is the characteristic finding. The nodules can range in size up to 3 cm, but most are less than 1 cm in diameter and are usually found in relation to small and medium-sized vessels and bronchi. This form of presentation may appear in many lung diseases and is easily mistaken for metastatic carcinoma, which is usually the initial radiologic interpretation [50]. However, little growth is shown on serial chest CT examinations [8, 50]. When this pattern is found the differential diagnosis list generally includes the following: pulmonary metastases, miliary granulomatous infection, sarcoidosis, silicosis, primary lung malignancy, and lymphangitic carcinomatosis.

There is a trend toward better prognosis for the multinodular pattern and the largest studies/reviews that assess the relation between imaging features/different patterns and prognosis show that longer survival corresponds to the presence of multiple pulmonary nodules [26–28, 30, 32].

To the best of our knowledge, at least when the imaging pattern was specifically reported, all the cases that report complete/partial response or with spontaneous regression correspond to the multinodular pattern [1, 27, 34, 37, 51–61]

This issue is extremely important because, due to the heterogeneity of PEH, the specific imaging pattern from radiologic diagnosis may be a pivot step in the approach of the patient with PEH and ultimately can define the option about the treatment. Furthermore, perhaps the results about treatment efficacy cannot be compared when the lesions do not have homogeneous patterns.

Particularly interesting is the fact already described in other tumors not so distinct from PEH, like is the case of bronchioloalveolar carcinoma. In these tumors it is the clinical pattern and pathologic stage, and not the degree of invasion on histologic examination, that can predict survival [62].

Multiple pulmonary reticulonodular opacities represent, histopathologically, areas of infiltrating nodular proliferation of neoplastic cells within the lumina of small blood vessels and lymphatic vessels [26, 29, 47, 49]. In fact, in their study, Woo et al. [32] found a clinical significance in their similarity to pulmonary metastases in terms of prognosis, although just three patients had this pattern. This pattern may also suggest a more aggressive infiltrative growth into the lung interstitium, depicted histopathologically by infiltrative nodular proliferation of tumor cells within the vessel lumina [63, 64].

When the disease presents as diffuse infiltrative pleural thickening with associated pleural effusion, it can simulate malignant pleural mesothelioma or diffuse pleural carcinomatosis [16, 45, 65, 66].

An extra pattern can be added to the previous three identified ones, defined as parenchymal tumor with pleural invasion [32]. This pattern was previously described in some reports in the literature [27, 67–70] and Woo et al. described it in 4 patients. The major importance of this pattern relies on the fact that imaging features can greatly simulate the much more common primary non-small-cell lung tumor. Indeed, as was the case with all the four patients of Woo et al. study, also in one of our patients primary lung cancer with pleural seeding was the initial differential diagnosis.

Other unusual radiologic findings in sporadic cases are described in the literature, like irregular interstitial thickening and ground-glass opacities as the dominant feature, mimicking diffuse lung disease [63].

Although histologic calcifications are common, radiologic visible calcifications are rare. In long-standing cases or after treatment, extensive calcification of the nodules can be seen [67, 71].

PET/CT findings can demonstrate increased FDG-uptakes but usually are not a pivotal tool for the diagnosis, and a negative PET cannot exclude PEH [72, 73]. This false negative result is thought to be caused because of the small size of nodules, little FDG activity in the neoplasm, or low cellular density. Conversely, other studies show that FDG uptake may reflect the activation of PEH tumor cells, which is a sign of a poorer prognosis [74, 75]. One particular study demonstrated the PET/CT scan utility to differentiate PEH from amyloidosis in a patient with both conditions [73]. Most patients who underwent PET/CT in the study by Woo et al. [32] showed increased FDG uptake at the corresponding lung lesions on CT.

The most problematic pattern, undoubtedly, is the multinodular pattern where the lung nodules were 20 mm or less in diameter, decreasing the diagnostic accuracy and reliability of PET-CT.

Due to its rarity and heterogeneous mode of presentation, diagnosis of PEH can be really defiant. The diagnosis usually requires a lung biopsy, and histopathological samples are usually obtained from open-lung or thoracoscopic biopsy. Chest CT-guided lung biopsy can also be an option due to potential increased risk of bleeding associated with this vascular-like tumor or when the lesion is peripheral. A few reports report diagnosis made through bronchoscopy biopsies [8, 71, 76]. Fine needle aspiration can produce erroneous results [77].

The diagnosis is made on the basis of these histopathological features and confirmed by positive immunohistochemistry staining for vascular-endothelial markers, like CD31, CD34, and factor VIII [46].

Some histologic findings, like spindle tumor cells and fibrinous pleuritic lesion with extrapleural tumor cells proliferation, are linked to a worse prognosis [27, 28, 78]. Regarding correlation between the histopathological findings and prognosis Kitaichi et al. [27] found that fibrinofibrous pleuritic lesions with extrapleural proliferation of tumor cells and the presence of spindle tumor cells were the characteristics associated with worse prognosis. Interestingly, intravascular invasion and invasion of the visceral pleura by tumor cells without fibrinous pleuritis were observed in the two patients with spontaneous partial regression in their study.

In a more recent study, Anderson et al. [14], in 39 cases of EH with thoracic involvement, found that higher tumor grade and the presence of pleural involvement in lung and/or pleural tumors correlate with poor prognosis. They suggest that the reported criteria for classifying and grading malignant vascular tumors within soft tissue might be applicable to tumors in the thoracic cavity, including PEH.

There is no established standard treatment consensus for therapeutic regimens available, particularly owing to its borderline malignancy features and scarcity of cases [30, 79]. Spontaneous partial regressions are reported, particularly in asymptomatic patients, as we already reported [27]. Radiotherapy has proven to be ineffective for PEH because of the tumor's radiobiological characteristics, particularly the slow growth of tumor cells [37]. Surgery when tumor resection is feasible is usually considered the treatment of choice [80, 81]. However, a complete surgical resection is usually not feasible. Chemotherapy/immunostimulants in patients with disseminated disease and lung transplantation can also be an option [28, 58, 59, 61, 81–85]. Although there are a small number of reports about the effectiveness of chemotherapy, a general standard of chemotherapy is usually accepted for PEH [8]. Pinet et al. [82] reported a case of an aggressive form of pleural EH resulting in complete remission after treatment with carboplatin plus etoposide. Given the vascular origin of this tumor, the use of antiangiogenetic drugs reasonably might achieve good outcomes, including thalidomide, lenalidomide, and bevacizumab [61, 79, 84–86]. Ye et al. [58], in their review concerning chemotherapy and immunotherapy used in PEH, demonstrated a good partial response to chemotherapy with carboplatin, paclitaxel, bevacizumab, thalidomide, and *α*-interferon. In their study, these authors also described three patients with stabilization of the disease and a dramatic improvement in clinical status when treated with a combination regimen that included Endostar® or bevacizumab. This was the first report of the efficacy of Endostar, a recently introduced recombinant human endostatin, considered to be a valuable antiangiogenic agent. However, no change in the size of the pulmonary nodules over the period of chemotherapy was observed and treatment was unable to stop the progression of disease. Further studies of treatment are warranted to provide a better knowledge for different combination chemotherapies options to treat PEH.

Regular follow-up with no active therapy has been employed in asymptomatic patients, specifically in cases with diffuse lesions corresponding to multinodular pattern [2, 74, 87].

## 5. Conclusions

These two cases emphasise the large spectrum of presentation, clinical behaviour, and outcome of PHE.

Further investigations are essential to elucidate the progression of the disease and therapeutic options for patients with PEH.

Particularly, different imaging patterns of PHE need to be correlated with the recent molecular/genetic features identified, in order to confirm their prognostic value.
